# The development of an adaptive upper-limb stroke rehabilitation robotic system

**DOI:** 10.1186/1743-0003-8-33

**Published:** 2011-06-16

**Authors:** Patricia Kan, Rajibul Huq, Jesse Hoey, Robby Goetschalckx, Alex Mihailidis

**Affiliations:** 1 Institute of Biomaterials and Biomedical Engineering, Rosebrugh Building, 164 College Street, Room 407, University of Toronto, Toronto, M5T 1P7, Canada; 2 School of Computing, University of Dundee, Dundee, DD1 4HN, UK; 3 Department of Occupational Science and Occupational Therapy, University of Toronto, 160-500 University Avenue, Toronto, M5G 1V7, Canada; 4 Toronto Rehabilitation Institute, 550 University Avenue, M5G 2A2, Toronto, Canada

## Abstract

**Background:**

Stroke is the primary cause of adult disability. To support this large population in recovery, robotic technologies are being developed to assist in the delivery of rehabilitation. This paper presents an automated system for a rehabilitation robotic device that guides stroke patients through an upper-limb reaching task. The system uses a decision theoretic model (a partially observable Markov decision process, or POMDP) as its primary engine for decision making. The POMDP allows the system to automatically modify exercise parameters to account for the specific needs and abilities of different individuals, and to use these parameters to take appropriate decisions about stroke rehabilitation exercises.

**Methods:**

The performance of the system was evaluated by comparing the decisions made by the system with those of a human therapist. A single patient participant was paired up with a therapist participant for the duration of the study, for a total of six sessions. Each session was an hour long and occurred three times a week for two weeks. During each session, three steps were followed: (A) after the system made a decision, the therapist either agreed or disagreed with the decision made; (B) the researcher had the device execute the decision made by the therapist; (C) the patient then performed the reaching exercise. These parts were repeated in the order of A-B-C until the end of the session. Qualitative and quantitative question were asked at the end of each session and at the completion of the study for both participants.

**Results:**

Overall, the therapist agreed with the system decisions approximately 65% of the time. In general, the therapist thought the system decisions were believable and could envision this system being used in both a clinical and home setting. The patient was satisfied with the system and would use this system as his/her primary method of rehabilitation.

**Conclusions:**

The data collected in this study can only be used to provide insight into the performance of the system since the sample size was limited. The next stage for this project is to test the system with a larger sample size to obtain significant results.

## Background

Stroke is the leading cause of physical disability and third leading cause of death in most countries around the world, including Canada [1] and the United States [2]. The consequences of stroke are devastating with approximately 75% of stroke sufferers being left with a permanent disability [3].

Research has shown that stroke rehabilitation can reduce the impairments and disabilities that are caused by stroke, and improve motor function, allowing stroke patients to regain much of their independence and quality of life. It is generally agreed that intensive, repetitive, and goal-directed rehabilitation improves motor function and cortical reorganization in stroke patients with both acute and long-term (chronic) impairments [4]. However, this recovery process is typically slow and labor-intensive, usually involving extensive interaction between one or more therapists and one patient. One of the main motivations for developing rehabilitation robotic devices is to automate interventions that are normally repetitive and physically demanding. These robots can provide stroke patients with intensive and reproducible movement training in time-unlimited durations, which can alleviate strain on therapists. In addition, these devices can provide therapists with accurate measures on patient performance and function (e.g. range of motion, speed, smoothness) during a therapeutic intervention, and also provide quantitative diagnosis and assessments of motor impairments such as spasticity, tone, and strength [5]. This technology makes it possible for a single therapist to supervise multiple patients simultaneously, which can contribute in the reduction of health care costs.

### Current upper-limb rehabilitation robotic devices

The upper extremities are typically affected more than the lower extremities after stroke [6]. Stroke patients with an affected upper-limb have difficulties performing many activities of daily living, such as reaching to grasp objects.

There have been several types of robotic devices designed to deliver upper-limb rehabilitation for people with paralyzed upper extremities. The Assisted Rehabilitation and Measurement (ARM) Guide [7] was designed to mimic the reaching motion. It consists of a single motor and chain drive that is used to move the user's hand along a linear constraint, which can be manually oriented in different angles to allow reaching in various directions. The ARM Guide implements a technique called "active assist therapy", in which its essential principle is to complete a desired movement for the user if they are unable to do so. The Mirror Image Movement Enabler (MIME) therapy system [8] consists of a six-degree of freedom (DOF) robot manipulator, which is attached to the orthosis supporting the user's affected arm. It applies forces to the limb during both unimanual and bimanual goal-directed movements in 3-dimensional (3D) space. Unilateral movements involve the robot moving or assisting the paretic limb towards a target in pre-programmed trajectories. The bimanual mode works in a slave configuration where the robot-assisted affected limb mirrors the unimpaired arm movements. The GENTLE/s system [9] is comprised of a commercially available 3-DOF robot, the HapticMASTER (FCS Robotics Inc.), which is attached to a wrist splint via a passive gimbal mechanism with 3-DOF. The gimbal allows for pronation/supination of the elbow as well as flexion and extension of the wrist. The seated user, whose arm is suspended from a sling to eliminate gravity effects, can perform reaching movements through interaction with the virtual environment on the computer screen. The rehabilitation robotic device that has received the most clinical testing is the Massachusetts Institute of Technology (MIT)-MANUS [10]. The MIT- MANUS consists of a 2-DOF robot manipulator that assists shoulder and elbow movements by moving the user's hand in the horizontal plane. Studies evaluating the effect of robotic therapy with the MIT-Manus in reducing chronic motor impairments show that there were statistically significant improvements in motor function [11-13]. The most recent study concluded that after nine months of robotic therapy, stroke patients with long-term impairments of the upper-limb improved in motor function compared with conventional therapy, but not with intensive therapy [14].

Recent work has attempted to make stroke rehabilitation exercises more relevant to real-life situations, by programming virtual reality games that mimic such situations (e.g. cooking, ironing, painting). The T-WREX system is one such attempt, an online Java-based set of exercises that can be combined with a stroke rehabilitation device such as the one described here [15]. Recent work has attempted to combine T-WREX with a non-invasive gesture exercise program based on computer vision. A user is observed with a camera, and his/her gestures are modeled and mapped into the T-WREX games. The user's progress can be monitored and reported to a therapist [16]. The work presented in [17] integrates virtual reality with robot assisted 3D haptic system for rehabilitation of children with hemiparetic cerebral palsy.

Researchers in the artificial intelligence community have started to design robot-assisted rehabilitation devices that implement artificial intelligence methods to improve upon the active assistance techniques found in the previous systems mentioned above. However, very few have been developed. An elbow and shoulder rehabilitation robot [18] was developed using a hybrid position/force fuzzy logic controller to assist the user's arm along predetermined linear or circular trajectories with specified loads. The robot helps to constrain the movements in the desired direction, if the user deviates from the predetermined path. Fuzzy logic was incorporated in the position and force control algorithms to cope with the nonlinear dynamics (i.e. uncertainty of the dynamics model of the user) of the robotic system to ensure operation for different users. An artificial neural network (ANN) based proportional-integral (PI) gain scheduling direct force controller [19] was developed to provide robotic assistance for upper extremity rehabilitation. The controller has the ability to automatically select appropriate PI gains to accommodate a wide range of users with varying physical conditions by training the ANN with estimated human arm parameters. The idea is to automatically tune the gains of the force controller based on the condition of each patient's arm parameters in order for it to apply the desired assistive force in an efficient and precise manner.

There exist several control approaches for robot assisted rehabilitation [20], however, most of them are devoted to modeling and prediction of the patients' motion trajectory and assisting them to complete the desired task. The work presented in [21] also proposes an adaptive system that provides minimum assistance to complete the desired task of the patients. While these robotic systems have shown promising results, none of them is able to provide an autonomous rehabilitation regime that accounts for the specific needs and abilities of each individual. Each user progresses in different ways and thus, exercises must be tailored to each individual differently. For example, the difficulty of an exercise should increase faster for those who are progressing well compared to those who are having trouble performing the exercise. The GENTLE/s system requires the user or therapist to constantly press a button in order for the system to be in operational mode [9]. It is imperative that a rehabilitation system operates with no or very little feedback as any direct input from the therapist (or user), such as setting a particular resistance level, prevents the user from performing the exercise uninterrupted. The system should be able to autonomously adjust different exercise parameters in accordance to each individual's needs. The rehabilitation systems discussed above also do not account for physiological factors, such as fatigue, which can have a significant impact on rehabilitation progress [22]. A system that can incorporate and estimate user fatigue can provide information as to when the user should take a break and rest, which may benefit rehabilitation progress.

The research described in this paper aims to fill these existing gaps by using stochastic modelling and decision theoretic reasoning to autonomously facilitate upper-limb reaching rehabilitation for moderate level stroke patients, tailor the exercise parameters for each individual, and estimate user fatigue. This paper will present a new controller that was developed based on a POMDP (partially observable Markov decision process), as well as early pilot data collected to show the efficacy of the new system.

## Rehabilitation system overview

**Figure 1:**
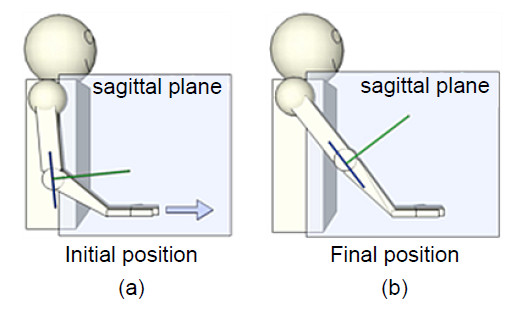
**The reaching exercise**. Starting from an initial position (a), the reaching exercise consists of a forward extension of the arm until it reaches the final position (b), then the return path brings the arm back to the initial position.

**Figure 2:**
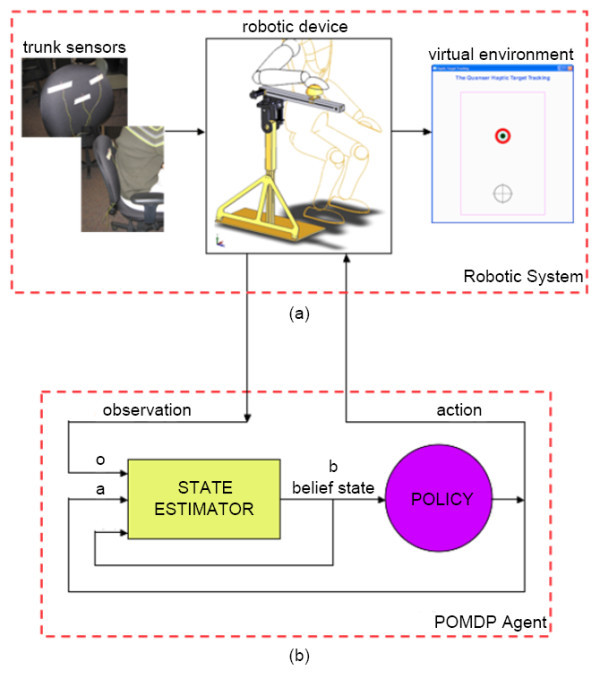
**Diagram of the reaching rehabilitation system**. The reaching rehabilitation system consists of the robotic system (a) and POMDP agent (b). The robotic system automates the reaching exercise and captures compensatory events. The POMDP system is the decision-maker of the system.

The automated upper-limb stroke rehabilitation system consists of three main components: the exercise (Figure 1), the robotic system (Figure 2a), and the POMDP agent (Figure 2b). As the user performs the reaching exercise on the robot, data from the robotic system are used as input to the POMDP, which decides on the next action for the system to take.

### The exercise

A targeted, load-bearing, forward reaching exercise was chosen for this project. Discussions with experienced occupational and physical therapists (n = 7) in a large rehabilitation hospital (Toronto, Canada) identified that this is an area of rehabilitation that is in need of more efficient tools. Moreover, reaching is one of the most important abilities to possess, as it is the basic motion involved in many activities of daily living. Figure 1 provides an overview of the reaching exercise. The reaching exercise is performed in the sagittal plane (aligned with the shoulder) and begins with a slight forward flexion of the shoulder, and extension of the elbow and wrist (Figure 1a). Weight is translated through the heel of the hand as it is pushed forward in the direction indicated by the arrow, until it reaches the final position (Figure 1b). The return path brings the arm back to the initial position. Therapists usually apply resistive forces (to emulate load- or weight-bearing) during the reaching exercise to strengthen the triceps and scapula musculature, which will help to provide postural support and anchoring for other body movements [23]. It is important to note that a proper reaching exercise is performed with control (e.g. no deviation from the straight path) and without compensation (e.g. trunk rotation, shoulder abduction/internal rotation).

The general progression during conventional reaching rehabilitation is to gradually increase target distance, and then to increase the resistance level, as indicated by one of the consulting therapists on this project. If patients are showing signs of fatigue during the exercise, therapists will typically let patients rest for a few minutes and then continue with the therapy session. The goal is to have patients successfully reach the furthest target at maximum resistance, while performing the exercise with control and proper posture.

### Robotic system

A novel robotic system (Figure 2a) was designed to automate the reaching exercise as well as to capture any compensatory events. The system is comprised of three main components: the robotic device, which emulates the load-bearing reaching exercise with haptic feedback, the postural sensors, which identify abnormalities in the upper extremities during the exercise, and the virtual environment, which provides the user with visual feedback of the exercise on a computer monitor.

**Figure 3:**
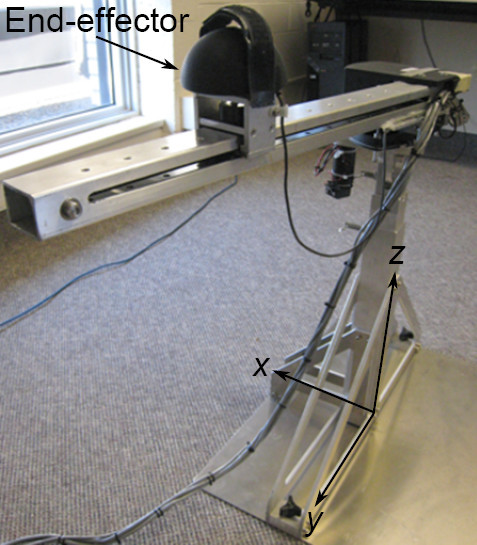
**Actual robotic rehabilitation device**. The robotic rehabilitation device features a non-restraining platform and allows the reaching exercise to be performed in 3D space.

The robotic device, as detailed in [24] and shown in Figure 3, was built by Quanser Inc., a robotics company in Toronto. It features a non-restraining platform for better usability and freedom of movement, and has two degrees of freedom, which allow the reaching exercise to be performed in 2D space. The robotic device also incorporates haptic technology, which provides feedback through sense of touch. For the purpose of this research, the haptic device provided resistance and boundary guidance for the user during the exercise, which was performed only in 2D space (in the horizontal plane parallel to the floor). Encoders in the end-effector of the robotic device provide data to indicate hand position and shoulder abduction/internal rotation (i.e. compensation) during the exercise.

**Figure 4:**
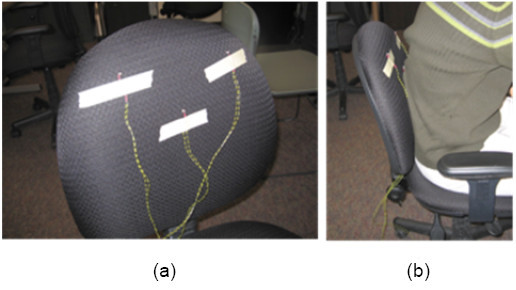
**Trunk photoresistor sensors**. The trunk photoresistor sensors are placed in three locations: lower back, lower left scapula, and lower right scapula (a). The detection of light indicates trunk rotation compensation (b).

The unobtrusive trunk sensors (Figure 4) provide data to indicate trunk rotation compensation. The trunk sensors are comprised of three photoresistors taped to the back of a chair, each in one of three locations: the lower back, lower left scapula, and lower right scapula. The detection of light during the exercise indicates trunk rotation, as it means a gap is present between the chair and user. Finally, the virtual environment provides the user with visual feedback on hand position and target location during the exercise. The reaching exercise is represented in the form of a 2D bull's eye game. The goal of the game is for the user to move the robot end-effector, which corresponds to the cross-tracker in the virtual environment, to the bull's eye target. The rectangular box is the virtual (haptic) boundary, which keeps the cross-tracker within those walls during the exercise.

### POMDP agent

The POMDP agent (Figure 2b) is the decision-maker of the system. Observation data from the robotic device is passed to a state estimator that estimates the progress of the user as a probability distribution over the possible states, known as a belief state. A policy then maps the belief state to an action for the system to execute, which can be either setting a new target position and resistance level or stopping the exercise. The goal of the POMDP agent is to help patients regain his/her maximum reaching distance at the most difficult level of resistance, while performing the exercises with control and proper posture.

## Partially observable Markov decision process

A POMDP is a decision-theoretic model that provides a natural framework for modeling complex planning problems with partial observability, uncertain action effects, incomplete knowledge of the state of the environment, and multiple interacting objectives. POMDPs are defined by: a finite set of world states *S*; a finite set of actions *A*; a finite set of observations *O*; a transition function *T *: *S × A *→ ∏(*S*), where ∏(*S*) denotes a probability distribution over states *S*, and P(*s'*|*s*,*a*) denotes the probability of transition from state *s *to *s' *when action *a *is performed; an observation function *Z *: *S × A *→ ∏(*O*), with P(*o*|*a*,*s'*) denoting the probability of observing *o *after performing action *a *and transiting to state *s'*; and a reward function *R *: *S × A × 0 *→ ℝ, with R(*s*,*o,a*) denoting the expected reward or cost (i.e. negative reward) incurred after performing action *a *and observing *o *in state *s*.

The POMDP agent is used to find a policy (i.e. course of action) that maximizes the expected discounted sum of rewards attained by the system over an infinite horizon, to monitor beliefs about the system state in real time, and to use the computed policy to decide which actions to take based on the belief states. For an overview of POMDPs, refer to [25,26].

### Examples of POMDPs in real-world applications

An increasing number of researchers in various fields are becoming interested in the application of POMDPs because they have shown promise in solving real-world problems.

Researchers at Carnegie Mellon University used a POMDP to model the high-level controller for an intelligent robot, Nursebot, designed to assist elderly individuals with mild cognitive and physical impairments in their daily activities such as taking medications, attending appointments, eating, drinking, bathing, and toileting [27]. Using variables such as the robot location, the user's location, and the user's status, the robot would decide whether to take an action, to provide the user a reminder or to guide the user where to move. By maintaining an accurate model of the user's daily plans and tracking his/her execution of the plans by observation, the robot could adapt to the user's behavior and take decisions about whether and when it was most appropriate to issue reminders.

A POMDP model was also used in a guidance system to assist people with dementia during the handwashing task [28]. By tracking the positions of the user's hands and towel with a camera mounted above the sink, the system could estimate the progress of the user during the handwashing task and provide assistance with the next step, if needed. Assistance was given in the form of verbal and/or visual prompts, or through the enlistment of a human caregiver's help. An important feature of this system is the ability to estimate and adapt to user states such as awareness, responsiveness, and overall dementia level which affect the amount of assistance given to the user during the handwashing activity.

### Justification for using a POMDP to model reaching rehabilitation

Classical planning generally consists of agents which operate in environments that are fully observable, deterministic, static, and discrete. Although these techniques can solve increasingly large state-space problems, they are not suitable for most robotic applications, such as the reaching task in upper-limb rehabilitation, as they usually have partial observability, stochastic actions, and dynamic environments [29]. Planning under uncertainty aims to improve robustness by factoring in the types of uncertainties that can occur. A POMDP is perhaps the most general representation for (single-agent) planning under uncertainty. It surpasses other techniques in terms of representational power because it can combine many important aspects for planning under uncertainty as described below.

In reality, the state of the world cannot be known with certainty due to inaccurate measurements from noisy and imperfect sensors, or instances where observations may be impossible and inferences must be made, such as the fatigue state of the patient. POMDPs can handle this uncertainty in state observability by expressing the state of the world as a belief state - the probability distribution over all possible states of the world - rather than actual world states. By capturing this uncertainty in the model, the POMDP has the ability to make better decisions than fully observable techniques. For example, the reaching rehabilitation system does not consist of physical sensors that can detect user fatigue. By capturing observations in user compensation and control, POMDPs can use this information to infer or estimate how fatigued the user is. Fully observable methods cannot capture user fatigue in this way since it is impossible to observe fatigue, unless it is physically captured such as using electrical stimulation to measure muscle contractions [30]. However, these techniques are invasive and may not even guarantee full observability of the world state since sensor measurements may be inaccurate.

The reaching exercise is a stochastic (dynamic) decision problem where there is uncertainty in the outcome of actions and the environment is always changing. Thus, choosing a particular action at a particular state does not always produce the same results. Instead, the action has a random chance of producing a specific result with a known probability. POMDPs can account for the realistic uncertainty of action effects in the decision process through its transition probabilities and reward function. By knowing the probabilities and rewards of the outcomes of taking an action in a specific state, the POMDP agent can estimate the likelihood of future outcomes to determine the optimal course of action to take in the present. This ability to consider the future effects of current actions allows the POMDP to trade off between alternative ways to satisfy a goal and plan for multiple interacting goals. It also allows the agent to build a policy that is capable of handling unexpected outcomes more robustly than many classical planners.

Different stroke patients progress in different ways during rehabilitation depending on their ability and state of health. It is imperative for the rehabilitation system to be able to tailor and adapt to each individual's needs and abilities over time. POMDPs have the capability of incorporating user abilities autonomously in real-time by keeping track of which actions have been observed to be the most effective in the past. For example, the POMDP may decide to keep the target closer for a longer period of time for patients who are progressing slowly, but may increase the target location further at a quicker rate for those who are progressing faster.

Since one of the objectives of a rehabilitation robotic system is to reduce health care costs by having one therapist supervise multiple stroke patients simultaneously, it is imperative to design the system in which no or very little explicit feedback from the therapist is required during the therapy session. The system must be able to effectively guide the patient during the reaching exercise without the need for explicit input (e.g. a button press to set a particular resistance level), as any direct input from the therapist would be time consuming and prevent the user from intensive repetition. POMDPs have this ability to operate autonomously through the estimation of states and then automatically making decisions. For eventually practising therapy in the home setting, it is especially important that the system does not require any explicit feedback since no therapist will be present.

## POMDP model

The specific POMDP model for the reaching exercise is described as follows.

### Actions, variables, and observations

**Figure 5:**
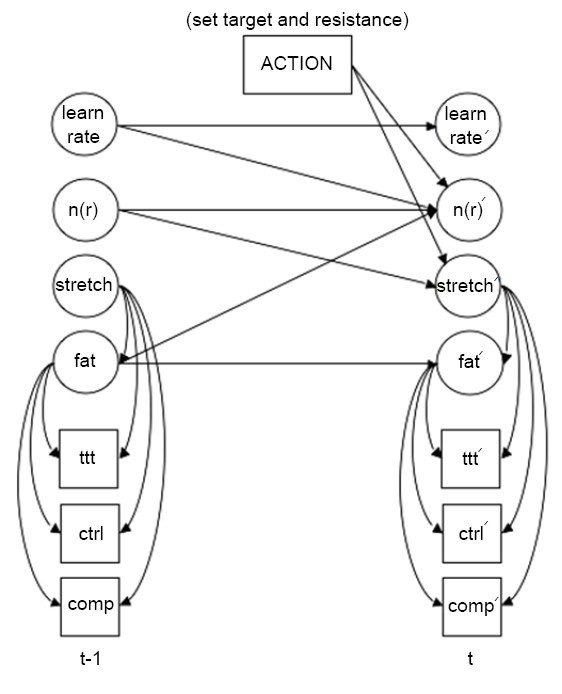
**POMDP model as a DBN**. The POMDP model consists of 7 state variables, 10 actions, and 3 observation variables. The arrows indicate how the variables at time *t-1 *influence those at time *t*. The variable *fatigue *is abbreviated as *fat*.

Figure 5 shows the POMDP model as a dynamic Bayesian network (DBN). There are 10 possible actions the system can take. These are comprised of nine actions of which each is a different combination of setting a target distance *d*∈{*d1*,*d2*,*d3*}, and resistance level *r*∈{*none*,*min*,*max*}, and one action to stop the exercise when the user is fatigued.

Variables were chosen to meaningfully capture the aspects of the reaching task that the system would require in order to effectively guide a stroke patient during the exercise. Unique combinations of instantiations of these variables represent all the different possible states of the rehabilitation exercise that the system could be in. The following variables were chosen to represent the exercise:

• *fatigue *= {*yes*,*no*} describes the user's level of fatigue

• *n*(*r*) = {*none*,*d1*,*d2*,*d3*} describes the range (or ability) of the user at a particular resistance level, *r*∈{*none*,*min*,*max*}. The range is defined as the furthest target distance, *d*∈{*d1*,*d2*,*d3*}, the user is able to reach at a particular resistance. For example, if *r *= *min *and the furthest target the user can reach is *d *= *d2*, then the user's range is *n*(*min*)=*d2*.

• *stretch *= {*+9*,*+8*,*+7*,*+6*,*+5*,*+4*,*+3*,*+2*,*+1*,*0*,*-1*,*-2*} describes the amount the system is asking the user to go beyond their current range. It is a deterministic function of the system's choice of resistance level (*a*_*r*_) and distance (*a*_*d*_), which measures how much this choice is going to push a user beyond their range, and is computed as follows:(1)

where *r *indexes the resistance level (with *1 *= *none*, *2 *= *min*, *3 *= *max*), *a*_*r*_*,a*_*d*_∈{*1*,*2*,*3*} index the resistance level and distance set by the system, and *n*_*r*_∈{*0*,*1*,*2*,*3*} indexes the range at *r*.

• *learnrate *= {*lo*,*med*,*hi*} describes how quickly the user is progressing during the exercise

The observations were chosen as follows:

• *ttt *= {*none*,*slow*,*norm*} describes the time it takes the user to reach the target

• *ctrl *= {*none*,*min*,*max*} describes the user's control level by their ability to stay on the straight path

• *comp *= {*yes*,*no*} describes any compensatory actions (i.e. improper posture) performed

Note that, although the observations are fully observable, the states are still not known with certainty since the fatigue, user range, stretch, and learning rate variables are unobservable and must be estimated.

### Dynamics

**Figure 6:**
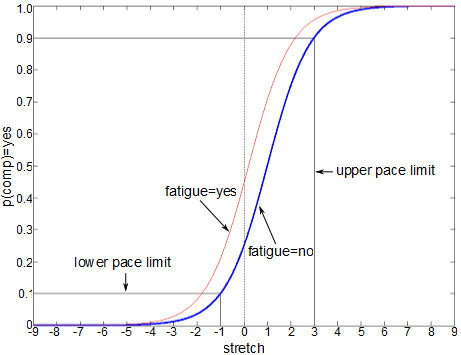
**Example pace function**. This is an example pace function for *comp *= *yes*. It shows the upper and lower pace limits, and the pace function for each condition of *fatigue *(abbreviated as *fat*).

The dynamics of all variables were specified manually using simple parametric functions of *stretch *and the user's *fatigue. *The functions relating *stretch *and *fatigue *levels to user performance are called *pace functions*. The pace function, *φ*, is a function of the stretch, *s*, and fatigue, *f*, and is a sigmoid function defined as follows:(2)

where *m *is the mean stretch (the value of stretch for which the function *φ *is 0.5 when the user is not fatigued), *m*(*f*) is a shift function that is dependent on the user's fatigue level (e.g. 0 if the user is not fatigued), and *σ*_*s *_is the slope of the pace function. There is one such pace function for each variable, and the value of the pace function at a particular stretch and fatigue level gives the probability of the variable in question being true in the following time step. Figure 6 shows an example of pace function for *comp = yes*. It shows that when the user is not fatigued and the system sets a target with a stretch of 3 (upper pace limit), the user might have a 90% chance to compensate. However, if the stretch is -1 (lower pace limit), then this chance might decrease to 10%. The pace limits decrease when the user is fatigued (at the same probability). In other words, the user is more likely to compensate when fatigued.

The detailed procedure of specifying *m*, *σ*_*s*_, and *m*(*f*) has been described in *Additional file *1 - *Pace function parameters*.

In the current model, the ranges *n*(*r*) were modeled separately, although they could also use the concept of pace functions. The dynamics for the ranges basically state that setting targets at or just above a user's range will cause their range to increase slowly, but less so if the user is fatigued. If a user's range is at *d3 *for a particular resistance, then practicing at that distance and resistance will increase their range at the next higher resistance from *none *to *d1*. The dynamics also includes constraints to ensure that ranges at higher resistances are always less than or equal to those at lower resistances. Finally, the dynamics of range include a dependency on the learning rate (*learnrate*): higher learning rates cause the ranges to increase more quickly.

### Rewards and computation

The reward function was constructed to motivate the system to guide the user to exercise at maximum target distance and resistance level, while performing the task with maximum control and without compensation. Thus, the system was given a large reward for getting the user to reach the furthest target distance (*d *= *d3*) at maximum resistance (*r *= *max*). Smaller rewards were given when targets were set at or above the user's current range (i.e. when *stretch *> = 0), and when the user was performing well (i.e. *ttt *= *norm*, *ctrl *= *max*, *comp *= *no*, and *fatigue *= *no*). However, no reward was given when the user was fatigued, failed to reach the target, had no control, or showed signs of compensation during the exercise. Please see Additional file 2 for the complete reward function of the model.

The POMDP model had 82,944 possible states. The size of this reaching rehabilitation model renders optimal solutions intractable, thus, an approximation method was used. This approximation technique exploits the structure of the large POMDP by first representing the model using algebraic decision diagrams (ADDs) and then employing a randomized point-based value iteration algorithm [31], which is based on the Perseus algorithm [32] with a bound on the size of the value function. The model was sampled with a set of 3,000 belief points that were generated through random simulation starting from 20 different initial belief states: one for every range possibility. The POMDP was solved on a dual AMD Opteron™ (2.4 GHz) CPU using a bound of 150 linear value functions and 150 iterations in approximately 13.96 hours.

### Simulation

A simulation program was developed in MATLAB^® ^(before user trials) to determine how well the model was performing in real-time. The performance of the POMDP model was subjectively rated by the researcher and focused on whether the system was making decisions in accordance to conventional reaching rehabilitation, which was: (i) gradually increasing target distance first, then resistance level as the user performed well (i.e. reached target in normal time, had maximum control, and did not compensate), and (ii) increasing the rate of fatigue if the user was not performing well (i.e. failed to reach the target, had no control, or compensated).

The simulation began with an initial belief state. The POMDP then decided on an action for the system to take, which was predetermined by the policy. Observation data was manually entered and a new belief state was computed. This cycle continued until the system stopped the exercise because the user was determined to be fatigued. Before the next cycle occurred, the simulation program reset the fatigue variable (i.e. user is un-fatigued after resting) and the user ranges were carried over.

Simulations performed on this model seemed to follow that of conventional reaching rehabilitation. During simulation, the POMDP slowly increased the target distance and resistance level when the user successfully reached the target in normal time, had maximum control, and did not compensate. However, once the user started to lose control, compensated, or had trouble reaching the target, the POMDP increased its belief that the user was fatigued and stopped the exercise to allow the user to rest. The following two examples illustrate the performance of the POMDP model.

**Figure 7:**
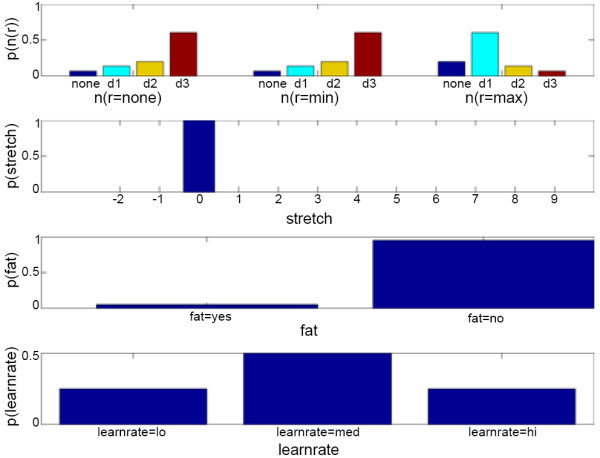
**Initial POMDP belief state of example 1**. This figure shows the initial belief state of *n*(*r*), *stretch*, *fatigue *(abbreviated as *fat*), and *learnrate*. The POMDP sets the target at *d *= *d1 *and resistance at *r *= *max*. The user reaches the target with *ttt *= *norm*, *ctrl *= *max*, and *comp *= *no*.

**Figure 8:**
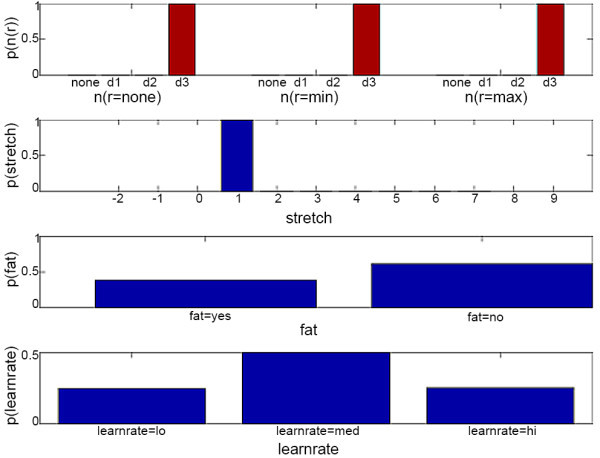
**Updated POMDP belief state of example 1**. This figure shows the updated belief state of *n*(*r*), *stretch*, *fatigue *(abbreviated as *fat*), and *learnrate *after the user compensates for the first time. The POMDP sets the target at *d *= *d3 *and resistance at *r *= *max*. The user reaches the target with *ttt *= *norm*, *ctrl *= *max*, and *comp *= *yes*.

Example 1 assumes that the user is able to reach the maximum target (*d *= *d3*) at the maximum resistance level (*r *= *max*), but then slowly starts to compensate after several repetitions. The initial belief state (Figure 7) assumes that the user's range at both zero and minimum resistance (i.e. *n*(*none*) and *n*(*min*)) is likely to be *d3*, and the user's range at maximum resistance (*n*(*max*)) is likely to be *d1*. In addition, the initial belief state assumes that the user is not fatigued with a 95% probability. From this belief state, the POMDP sets the first action to be *d *= *d1 *and *r *= *max*. According to the assumption, the user successfully reaches this target in normal time, with maximum control, and with no compensation. In the next five time steps, the POMDP sets the target at *d *= *d2 *and then increases it to *d *= *d3*, assuming the user successfully reaches each target with maximum control and no compensation. Here, the user's fatigue level has increased slowly from approximately 5% to 20% due to repetition of the exercise. Now, during the next time step when the POMDP decides to set the target at *d *= *d3 *again, the user compensates but is still able to reach the target with maximum control. Figure 8 shows the updated belief state. The fatigue level has jumped to about 40% due to user compensation. The POMDP sets the same target during the next time step and the user compensates once more. This time, the POMDP decides to stop the exercise because it believes the user is fatigued due to performing compensatory movements for two consecutive times. For the complete simulation, please see *Additional file *3 - *POMDP Simulation Example 1*.

**Figure 9:**
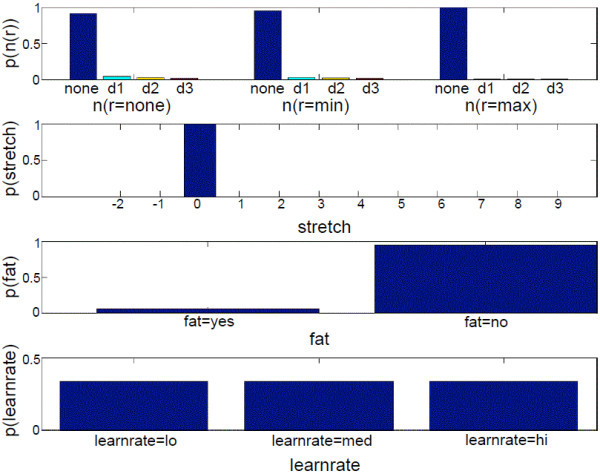
**Initial POMDP belief state of example 2**. This figure shows the initial belief state of *n*(*r*), *stretch*, *fatigue *(abbreviated as *fat*), and *learnrate*. The POMDP sets the target at *d *= *d1 *and resistance at *r *= none. The user reaches the target with *ttt *= *norm*, *ctrl *= *max*, and *comp *= *no*.

**Figure 10:**
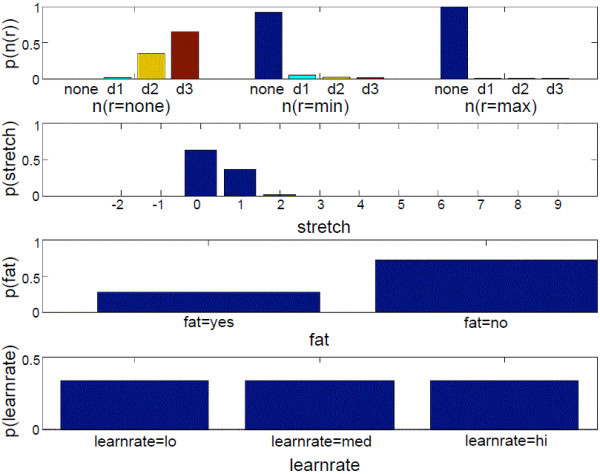
**Updated POMDP belief state of example 2**. This figure shows the updated belief state of *n*(*r*), *stretch*, *fatigue *(abbreviated as *fat*), and *learnrate *when the user failed to reach the target at *d *= *d3*. The POMDP resets the target at *d *= *d3 *and resistance at *r *= *none*. The user reaches the target with *ttt *= *norm*, *ctrl *=*max*, and *comp *= *no*.

In the second simulation example, the user is assumed to have trouble reaching the maximum target, *d *= *d3*, at zero resistance, *r *= *none*. The simulation starts with the initial belief state (shown in Figure 9), which assumes that the user's range at each resistance (i.e. *n*(*none*), *n*(*min*), and *n*(*max*)) is likely to be *none*, and that the user is not fatigued with a 95% probability. The POMDP slowly increases the target distance from *d1*, to *d2*, and then to *d3 *while keeping at the same resistance level (*r *= *none*) when the user successfully reaches the target in normal time, with maximum control, and with no compensation. However, at *d *= *d3 *the user fails to reach the target (i.e. *ttt *= *none*), has minimum control (*ctrl *= *min*), and does not compensate (*comp *= *no*). The updated belief state is shown in Figure 10, where the fatigue level jumped from about 10% to 25% due to the failure in reaching target. After the user failed to reach *d3*, the POMDP decides to keep the same target at *d3 *since *stretch *is about 75% likely to be 0 (i.e. at the user's range). Again, the user fails to reach the target with minimum control and no compensation and the level of fatigue increased to about 40%. The POMDP decides to stop the exercise when the user again failed to reach d3 and performed a compensatory movement. Hence, the fatigue level changed to about 60%. For the complete simulation, please see *Additional file *4 - *POMDP Simulation Example 2*.

## Pilot Study - Efficacy of POMDP

A pilot study was conduced with therapists and stroke patients to evaluate the efficacy of the POMDP agent - i.e. the correctness of the decisions being made by the system.

### Participants

Due to a delay in receiving ethics approval, only one therapist and one patient were recruited for the study. As such, several simulations were also run (as previously described and presented later in this section) to help draw conclusions regarding the efficacy of the POMDP. The therapist was a physical therapist with more than nine years of experience in post-acute upper-limb stroke rehabilitation, and was fluent in English. The patient was right-side hemiparetic, had a stroke onset of 227 days (7 months and 14 days) before enrolment, scored 4 on the arm section of the Chedoke-McMaster Stroke Assessment (CMSA) Scale [33], was able to move to some degree but still had impaired movements as determined by their therapist, and could understand and respond to simple instructions.

### Method

The patient participant was paired up with the therapist participant for the duration of the study. Each session lasted for approximately one hour and was completed three times a week for two weeks.

For each session, the therapist brought the patient to the testing room. The patient participant was seated on a regular, straight-back chair positioned to the left of the robotic device. The therapist was responsible for adjusting the position of the chair, placing the trunk sensors at the appropriate spots (lower back, lower left scapula, and lower right scapula), and adjusting the height of the robot to ensure that the end-effector was correctly positioned in the sagittal plane of the patient's right shoulder. When both participants were ready to begin, the researcher powered on the robotic device and started the computer programs that controlled the POMDP agent, robotic device, and virtual environment. The patient was asked to place his/her hand on the end-effector, which was secured with a comfortable strap, and when ready, the researcher set the initial belief state of the POMDP and started the exercise.

The exercise was performed in three parts: (A) after the POMDP made a decision (i.e. to set the target position and resistance level, or to stop the exercise) the therapist either agreed or disagreed with the decision made; (B) the researcher had the device either execute the decision made by the POMDP if the therapist agreed or execute the decision made by the therapist if the therapist disagreed; and (C) the patient then performed the reaching exercise by trying to reach the target on the computer screen. These parts were repeated in the order of A-B-C until the end of the session.

Questions were asked at the end of each session and at the completion of the study for both participants. The questionnaire for the therapist participant was designed to focus on rating the decision-making strategy of the POMDP agent. For the patient participant, the questionnaire focused on gathering feedback with respect to their satisfaction in using such a robotic system. Both questionnaires consisted of quantitative and qualitative questions for statistical analysis and to provide insight into future design improvements, respectively. A four-point Likert scale was used for each quantitative question, with 1 representing complete disagreement and 4 representing complete agreement.

## Results and discussion

The small sample size of the study limited the use of hypothesis testing to interpret the data. Thus, the data collected in the study from one therapist and one patient can only provide insight into the performance of the system. A more detailed study will be completed in the spring of 2010.

### Agreement of POMDP decisions

**Figure 11:**
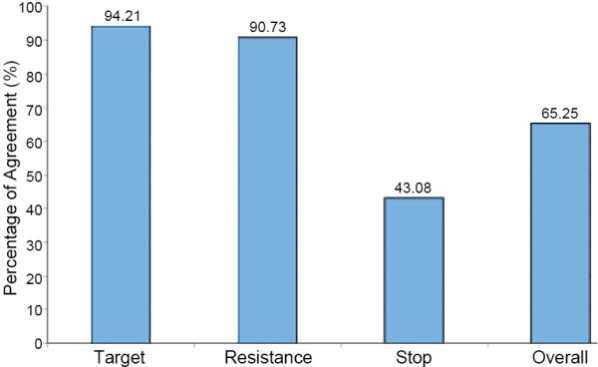
**Percentage of therapist agreement with POMDP**. This figure shows the percentage of therapist agreement with the decisions made by the POMDP on target distance, resistance level, and stopping the exercise, as well as the overall performance of the system, over all sessions.

Every decision made by both the POMDP and therapist was decomposed into three separate decisions: 1) the distance to set the target, 2) the level to set the resistance, and 3) whether or not to stop the exercise. The level of agreement by the therapist to the decisions made by the POMDP was calculated based on the three separate decisions as described above. A point of agreement would be given if the therapist set the same target distance as the POMDP, set the same resistance level as the POMDP, or agreed with the POMDP to stop the exercise or not. Figure 11 shows the percentage of agreement over all sessions. Note that there were 636 state transitions (i.e. total number of trials) and 1,154 decisions made during the study.

The therapist agreed with both the target distance and resistance level decisions made by the POMDP approximately 94% and 90% of the time, respectively, during the study (shown in Figure 9). Most of this agreement was with the POMDP repeatedly setting the target distance at *d3 *and the resistance at *max*. Since the patient was able to reach this setting within the first session with proper posture and control, the POMDP continued to make this decision as it was given large rewards for getting the user to reach the furthest target at maximum resistance. The therapist generally agreed with these decisions, as she wanted the patient to work on strengthening. It is important to note that this was a problem in the experimental design, where the mapping from the system resistance levels to the actual physical resistance in the device was not tuned properly for the user in the study. Before the trials began, therapists tested the system and concluded that the resistance levels were sufficient for moderate-level stroke patients. However, for future trials, initializing the resistance levels for different users should be properly developed based on some initial trials.

The therapists only agreed with the POMDP approximately 43% of the time for the stop decision. The POMDP wanted to stop the exercise to let the user take a break far more often than the therapist wanted. If the therapist did not see any signs of fatigue from the user, she would have the patient continue practising the exercise for a longer period of time and not stop. The dynamics of the fatigue variable in the POMDP model caused its progression to *fatigue *= *yes *too quickly. Decreasing this progression to match that of the therapist's decision of stopping the exercise can be fixed by adjusting the fatigue effects in the model. Since the percentage of agreement for the stop decision was low, the overall therapist agreement with the POMDP decisions dropped to approximately 65%.

During each session, as soon as the POMDP estimated that the patient was fatigued, it continually made the decision to stop the exercise no matter the decision the therapist entered into the system. That is, the POMDP would continue to call for a stop from the time it first did so until the therapist finally agreed. If the repeated stop decisions were discarded, the percentage of agreement would have been approximately 94%.

The therapist's decisions alternated between having the patient work on muscle strengthening (by repeatedly setting the distance and resistance at the highest level) and on control (by randomizing the target distance and resistance levels). However, randomization was not part of the POMDP's initial objective and thus, the POMDP would never make the decision to randomize the target distance and resistance levels.

### Questionnaire Data

**Figure 12:**
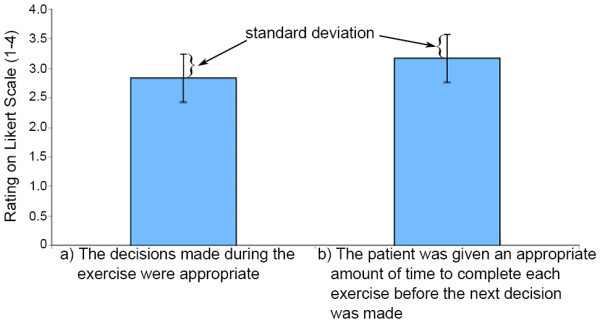
**Therapist evaluation on POMDP decisions**. This figure summarizes the evaluation of POMDP decisions made by the therapist on a Likert scale with a mean and SD of 2.833 and 0.408, respectively, for question (a) and a mean and SD of 3.167 and 0.408, respectively, for question (b).

Figure 12 summarizes the therapist's session responses, in terms of mean and standard deviation (SD), regarding the appropriateness of the decisions made during the exercise and whether the patient was given enough time to complete each exercise before the next decision was made. The therapist's rating on the appropriateness of the amount of time given to complete each exercise before the next decision was made was generally favorable with a mean score of 3.2 out of 4.0 on the Likert scale. However, the appropriateness of the decisions made by the POMDP during the sessions was less favorable with a mean score of 2.8 out of 4.0. Comments from the therapist suggested that randomizing the target distance and resistance level would be beneficial for the patient to work on control in addition to strengthening, which the POMDP did by repeatedly setting the target distance at *d3 *and the resistance level at *max *(once the patient was able to perform the exercise at these settings). The initial specification of the POMDP model was based on the repeated exercise for strengthening only, and did not include any utility function promoting the practice of control through randomization. This could be included in future versions by explicitly modeling the fact that a sequence of differing resistance and distance levels can improve a client's control.

In addition to the quantitative ratings in the session questionnaire, a qualitative question was asked to encourage the therapist to elaborate on any aspects related to the decisions made by the POMDP agent. The general qualitative results from the therapist for the final questionnaire can be summarized as follows: 1) the POMDP decisions were believable, except that the POMDP wanted to stop the exercise too early, and 2) the therapist could envision the rehabilitation system being used in both the clinic and home setting, as long as the system could vary the locations of the target and not restrict it to a straight path for more patient motivation, and was easy to set up for therapists.

With the help of a translator, the patient was able to answer the final questionnaire at the end of the study, which consisted of eight quantitative four-point Likert scale questions and four qualitative questions. From the patient's quantitative results, the patient found the quality of motion of the robotic device to be very smooth with a score of 4.0 out of 4.0. The patient also felt that the resistance applied by the robotic device was too little, scoring 1.0 out of 4.0. Throughout the study, the patient repeatedly commented that the exercise was "too easy", again a reflection of the device's resistance levels not being properly tuned to this particular user before the start of the trial. The patient was not able to feel the trunk sensors at all during the exercise, which suggests that trunk compensatory movements can be captured unobtrusively. The patient also felt that the bull's eye game was somewhat interesting, scoring 3.0 out of 4.0. The patient felt that the exercise closely resembled the reaching motion and conventional upper-limb therapy, scoring 3.0 out of 4.0 for both. In addition, the patient believed he would use this robotic system as his primary therapy, scoring 4.0 out of 4.0. The patient did not elaborate on the qualitative questions, thus, feedback from this section of the questionnaire was discarded.

## Future work

The immediate future work of this project is to test the POMDP model with more participants in order to obtain significant results. Besides this, the results from the pilot study provide the following insight into the future development of the POMDP model and overall system.

• The effect of randomization of different target distances and resistance levels on control needs to be studied.

**Figure 13:**
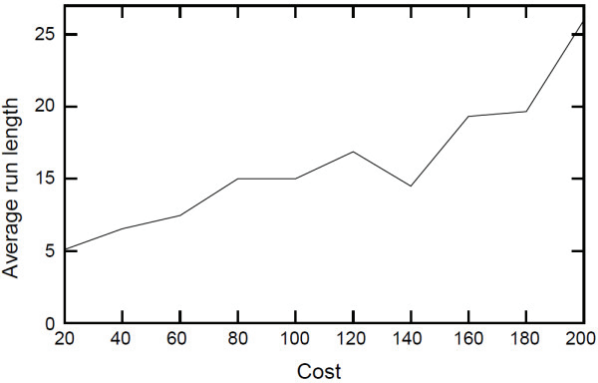
***Exercise run lengths for different costs of *stop**. This figure shows the average run length for different costs of the stop action. Increasing the cost of the *stop *generates, on average, longer runs.

**Figure 14:**
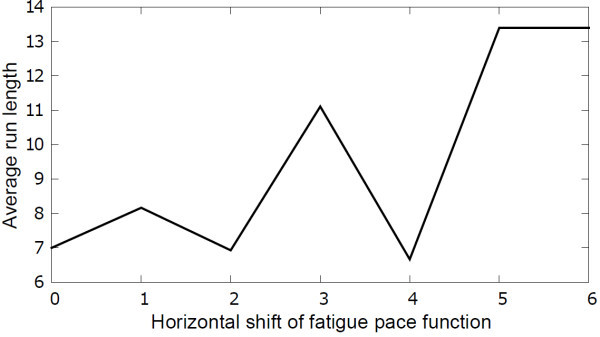
**Exercise run lengths for different shifts in *fatigue***. This figure shows the average run length for different horizontal shifts of the *fatigue *pace function. Lowering the probability of fatigue generates longer runs.

The dynamics of the fatigue variable and the cost of stop action may need to be changed in order to stop the exercise less often. This problem can be solved in various ways. To show this, two simulations were run - 1) with varying costs of the *stop *decision, and 2) with varying horizontal shift of fatigue pace function. The result of the first experiment is shown in Figure 13, which shows that increasing the cost of the stop action generates, on average, longer runs. The result of the second experiment is shown in Figure 14, which shows that having a lower probability of fatigue generates longer runs. A lower probability of fatigue is achieved by shifting the fatigue pace function horizontally. In this case, the system thinks that the user will be less likely to get fatigued for exercises with the same stretch. These simulated results overall demonstrate that the therapist can adjust the policy of interaction substantially, to suit their and their client's needs.

**Figure 15:**
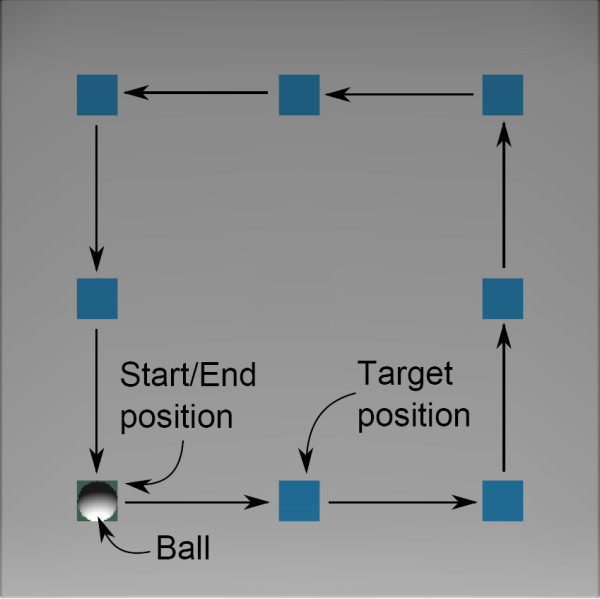
**2D reaching exercise**. This figure shows the virtual environment for 2D reaching exercise.

**Figure 16:**
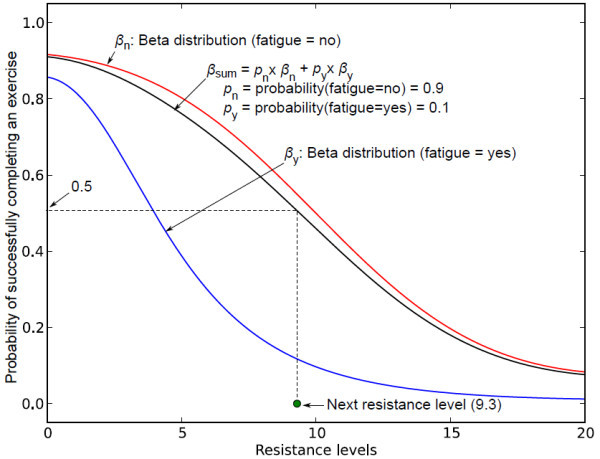
**Beta distribution**. This figure shows continuous action space using Beta distribution.

• The POMDP model needs to be expanded in order to include targets in 2D space. As a first step of this expansion, currently we are developing 2D virtual games that include target positions in 2D space. Figure 15 shows an example where the target positions are set in a rectangular trajectory and the reaching task is to position the ball, which represents the end-effector of the robot, in the designated target position.

• The current robotic system only applies three discrete levels of resistance, which can be either increased or maintained at the same level during the exercise. The system will be more realistic if it is able to select varying levels of resistance that can be both increased and decreased to cope up with the need of an individual patient. Decreasing the resistance level may also result in lower fatigue probability and less frequent compensatory motion, which in turn may lead to longer duration of the exercise. To include these features into the current system, we are currently formulating a new probabilistic framework that models the user ability using Beta distributions [34] as a function of continuous resistance levels. A Beta distribution is initially chosen since it is suitable for modeling success or failure in continuous space. Figure 16 shows a simulated example with a range of continuous resistance levels from 0-20, where the probability of successfully finishing an exercise at a given resistance level is modeled with the following Beta distributions: *β*_*n *_in case the person is not fatigued and *β*_*y *_in case the person is fatigued.

**Figure 17:**
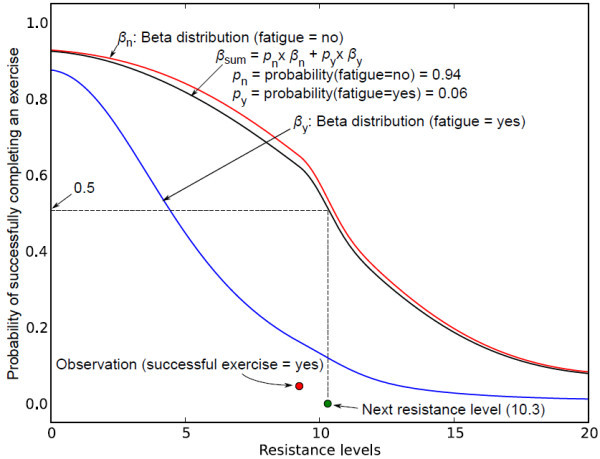
**Updated Beta distribution after the first observation**. This figure shows the updated Beta distribution after the first observation.

The total model is a weighted mixture of these two distributions, weighted according to the current belief that *fatigue = yes*. In this example, the posterior belief state assumes that probability of *fatigue *= *no *is 0.9 and probability of *fatigue *= *yes *is 0.1. The mixture model can be used to select the next resistance level for the exercise. In this example, the next resistance level 9.3 (shown in green circle in Figure 11) is selected as the maximum resistance that produces *β_sum _*≥ 0.5. Figure 10(a) shows the next sequence where the distributions and the belief state are updated using the simulated observation that the person successfully completed the exercise (shown in red circle in Figure 17) at the resistance level 9.3.

The updated model is the posterior according to Bayes' rule. The next resistance level is set to 10.3 according to the updated . Figure 10(b) shows an instance where the distributions and belief state is updated after five observations. The first four observations are successful exercises (shown in red circle in Figure 10(b)) and the last one is an unsuccessful exercise (the person did not reach the goal within acceptable time and control or had to compensate too much - shown in blue circle in Figure 10(b)). As a result, the next resistance level is set smaller compared to the current resistance. The exercise can be continued until the probability of *fatigue *= *yes *reaches a predefined threshold. Hence, this formulation - 1) is able to increase and decrease resistance levels in continuous space, and 2) is more adaptive to each individual patient's need since the distributions - the model of the person's abilities - are updated with the new observations. The initial shapes of the distributions can also be varied according to the condition of individual patient so that it produces appropriate resistance level while starting the exercise.

**Figure 18:**
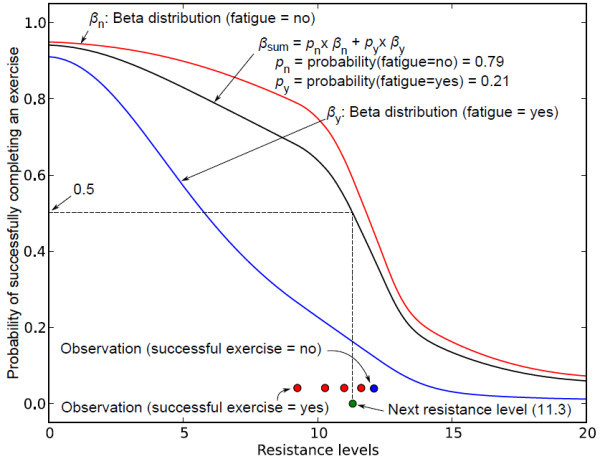
**Updated Beta distribution after five observations**. This figure shows the updated Beta distribution after five observations.

As shown in Figure 18, the same formulation can be applied to other state variables of the system. The preceding simulations are meant to demonstrate the feasibility of such a representation, and we are currently in the process of applying them to our rehabilitation device.

## Conclusions

This paper presents a POMDP system that is designed for an upper-limb rehabilitation robotic device. A POMDP was chosen for this system because it has the ability to handle partial observability (e.g. user fatigue), adapt to users' needs, and operate autonomously. The goal of the POMDP agent is to help patients regain their maximum reaching distance at the most difficult level of resistance, while performing the exercises with control and proper posture. Computer simulations of the POMDP model showed that the POMDP was making decisions in alignment to those of conventional reaching rehabilitation, which was to gradually increase target distance first, then resistance level as the user performed well, and increase the rate of fatigue if the user was not performing well.

The performance of the system was also evaluated by comparing the decisions made by the system with those of a human therapist. A single patient participant was paired up with a therapist participant for the duration of the study. Overall, the therapist agreed with the system decisions approximately 65% of the time. In general, the therapist thought the system decisions were believable and could envision this system being used in both a clinical and home setting. The patient was satisfied with the system and would use this system as her primary method of rehabilitation. The data collected in this study can only be used to provide insight into the performance of the system since the sample size was limited. As a result, the immediate future work of this project would be to test this POMDP model with more participants in order to obtain significant results.

The feedback from the therapist also suggests that the present system needs to include 2D target locations and varying levels of resistance. To include these features into the current system, we are currently developing virtual games with 2D target locations and a new probabilistic framework that expresses the probability of successfully completing an exercise using Beta distributions as a function of continuous resistance levels. The distributions are continuously updated with the new observations to reflect the performance of each individual patient. The system is also able to increase or decrease resistance levels according to the performance of a patient. The flexibility of decreasing resistance levels may also result in lower fatigue probability and thus may prevent early stopping of the exercise. The following suggestions of the therapist will also be considered in the future development:

• set mapping from resistance levels in the POMDP agent to actual resistance in the device for each user based on some initial trials,

• enhance the user interface to provide feedback for the user such as a scoring system or sounds to indicate that the user has reached the target, and

• develop an easier way to initialize the exercise such that all programs start automatically

Overall, this research demonstrates that POMDPs have promising potential to provide autonomous upper-limb rehabilitation for stroke patients, which may allow clients to perform guided rehabilitation when and where they prefer and enable them to progress at the best possible pace.

## Competing interests

The authors declare that they have no competing interests.

## Authors' contributions

PK and JH designed and developed the POMDP system. PK integrated the POMDP system with all aspects of the robotic system, developed and conducted the evaluation study of the overall integrated system, analyzed the study data, and drafted the manuscript. JH and RG conducted simulations post-trial to demonstrate how to solve the POMDP's early stopping problem. RH and RG added the simulation results of 2D virtual environment for reaching exercise and a new probabilistic framework that expresses the probability of successfully completing an exercise using Beta distributions as a function of continuous resistance levels. AM supervised the project. PK, JH, and AM participated in the conception and design of the POMDP system, and all authors read and approved the final manuscript.

## Supplementary Material

Additional file 1 **Pace functions parameters**. This file describes the procedure of specifying the parameters of a pace function. Click here for file

Additional file 2 **Reward function**. This file summarizes the reward function of the POMDP model. Click here for file

Additional file 3 **POMDP simulation example 1**. This file shows the simulation steps of example 1. Click here for file

Additional file 4 **POMDP simulation example 2**. This file shows the simulation steps of example 2. Click here for file
