# Supplementary material for: The development of an adaptive upper-limb stroke rehabilitation robotic system
Source: J Neuroeng Rehabil. 2011 Jun 16;8:33. doi: 10.1186/1743-0003-8-33 (PMC3152889; doi:10.1186/1743-0003-8-33)
Supplement: Additional file 1 — Pace functions parameters. This file describes the procedure of specifying the parameters of a pace function. [file 1743-0003-8-33-S1.DOC]

# Parameters of a pace function

The pace function, *φ*, is a function of the stretch, *s*, and fatigue, *f*, and is a sigmoid function defined as follows:

| $\varphi(s,f)=\frac{1}{1+e^{-\left[\frac{s-m-m(f)}{\sigma_{s}}\right]}},$ | (1) |
| --- | --- |

where *m* is the mean stretch (the value of stretch for which the function *φ* is 0.5 when the user is not fatigued), *m*(*f*) is a shift function that is dependent on the user’s fatigue level (e.g. 0 if the user is not fatigued), and *σ_s_* is the slope of the pace function. For each pace function, there are three parameters that needed to be specified: *m*, *σ_s_*, and *m*(*f*) (where the latter is technically a function, but for a binary fatigue variable such as we have, it is a single real-valued parameter). However, it is usually simpler to specify a pace function in terms of the upper and lower *pace limits*: the values of *stretch* for which a user’s performance will vary by a certain probability when the user is not fatigued (i.e. *m*(*f*)=*0*). For example, the *upper pace limit*, *s^+^*, for a user’s failure to reach a target (*ttt*=*none*) is the *stretch* at which the user will fail to reach the target with a probability of *φ^+^*. Similarly, the *lower pace limit*, *s^-^*, for *ttt*=*none* is the *stretch* at which the user will fail to reach the target with a probability of *φ^-^* (i.e. succeed in reaching it with probability *1-φ^-^*). Therefore, the following equations were derived:

| $\varphi^{+}=\frac{1}{1+e^{-(s^{+}-m)/\sigma_{s}}}$ | (2) |
| --- | --- |

| $\varphi^{-}=\frac{1}{1+e^{-(s^{-}-m)/\sigma_{s}}}$ | (3) |
| --- | --- |

, which could be solved for *m* and *σ_s_*.

Setting *pace limits* for the variables *ttt*, *ctrl*, and *comp* was simple and intuitive, but setting them for the user’s fatigue level was more problematic since it was difficult to quantify how much more fatigued a user gets in a single repetition. It is more intuitive to specify how many repetitions it takes for a user to become fatigued with a certain probability at some level of *stretch*. Since the pace function gives the probability of a user becoming fatigued in a single step (or repetition), we can compute the probability the user will be fatigued after some number of steps by multiplying the single-step probabilities together. Solving the resulting equations allowed us to specify the pace function parameters using the time intervals.

| 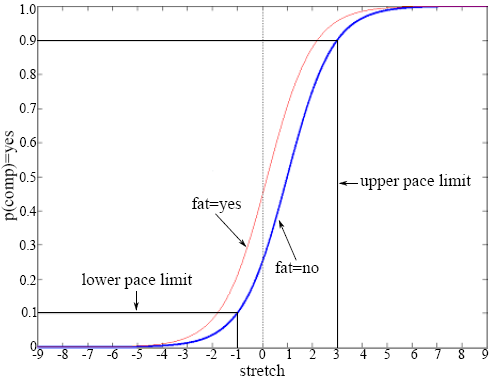 |
| --- |
| Figure 1 - Example pace function This is an example pace function for *comp*=*yes*, with *φ^+^*=0.9, *φ^-^*=0.1, *s^+^*=+3, *s^-^*=-1, *m*(*f*=*yes*)=0.8, and *m*(*f*=*no*)=0.0. It shows the upper and lower pace limits, and the pace function for each condition of *fatigue* (abbreviated as *fat*). |

The last parameter to specify was the fatigue effect *m*(*f*), which is a negative number that shifts the pace function downwards, as shown in Figure 1. The amount of shift indicates how much the *pace limits* will be shifted down when the user is fatigued. Thus, a user with an upper pace limit for *comp*=*yes* of *s^+^*=*3.0* and a fatigue effect of *m*(*f*=*yes*)=*0.8* will compensate with probability *φ^+^* if the target is set with a stretch of *3.0* when not fatigued, but will compensate with probability also *φ^+^* for a stretch of only *3.0-0.8*=*2.2* when fatigued. In other words, the user is more likely to compensate when fatigued, and the increase in probability of compensation is given by the fatigue effect, *m*(*f*). For variables with three values, such as *ctrl* and *ttt*, two pace functions need to be specified, one for the lowest value and one for the highest. The middle value gets what is left of the probability mass.
