# Supplementary material for: The development of an adaptive upper-limb stroke rehabilitation robotic system
Source: J Neuroeng Rehabil. 2011 Jun 16;8:33. doi: 10.1186/1743-0003-8-33 (PMC3152889; doi:10.1186/1743-0003-8-33)
Supplement: Additional file 2 — Reward function. This file summarizes the reward function of the POMDP model. [file 1743-0003-8-33-S2.DOC]

## Reward function of the POMDP model

| **Aspect** | **Definition** | **Reward Value** |
| --- | --- | --- |
| Larger rewards were given for setting r higher | *r*=none | 1 |
|  | *r*=min | 14 |
|  | *r*=max | 80 |
| Larger rewards were given for setting d higher | *d*=d1 | 1 |
|  | *d*=d2 | 9 |
|  | *d*=d3 | 11 |
| Larger rewards were given when user reached target in normal time | *ttt*=none | 0 |
|  | *ttt*=slow | 0.8 |
|  | *ttt*=norm | 1 |
| Larger rewards were given when user had control | *ctrl*=none | 0 |
|  | *ctrl*=min | 0.3 |
|  | *ctrl*=max | 1 |
| Reward was given when user did not compensate | *comp*=yes | 0 |
|  | *comp*=no | 1 |
| Reward was given when user was not fatigued | *fat*=yes | 0 |
|  | *fat*=no | 1 |
| Small rewards were given when d  and r were set at or above n(r); none were given when d and r were set below n(r) | *stretch*= -2 | 0 |
|  | *stretch* = -1 | 0 |
|  | *stretch* = 0 | 0.4 |
|  | *stretch* = +1 | 1 |
|  | *stretch* = +2 | 1 |
|  | *stretch* = +3 | 1 |
|  | *stretch* = +4 | 1 |
|  | *stretch* = +5 | 1 |
|  | *stretch* = +6 | 1 |
|  | *stretch* = +7 | 1 |
|  | *stretch* = +8 | 1 |
|  | *stretch* = +9 | 1 |
