# Supplementary material for: The development of an adaptive upper-limb stroke rehabilitation robotic system
Source: J Neuroeng Rehabil. 2011 Jun 16;8:33. doi: 10.1186/1743-0003-8-33 (PMC3152889; doi:10.1186/1743-0003-8-33)
Supplement: Additional file 3 — POMDP simulation example 1. This file shows the simulation steps of example 1. [file 1743-0003-8-33-S3.DOC]

# Simulation steps of Example 1

| Decision  Steps | Belief state | Actions &  observations |
| --- | --- | --- |
| Step 1 |  | *d*=d1  *r*=max  *ttt*=norm  *ctrl*=max  *comp*=no |
| Step 2 |  | *d* =d2  *r* =max  *ttt* =norm  *ctrl* =max  *comp* =no |
| Step 3 |  | *d* =d3  *r* =max  *ttt* =norm  *ctrl* =max  *comp* =no |
| Step 4 |  | *d* =d3  *r* =max  *ttt* =norm  *ctrl* =max  *comp* =no |
| Step 5 |  | *d* =d3  *r* =max  *ttt* =norm  *ctrl* =max  *comp* =no |
| Step 6 |  | *d* =d3  *r* =max  *ttt* =norm  *ctrl* =max  *comp* =no |
| Step 7 |  | *d* =d3  *r* =max  *ttt* =norm  *ctrl* =max  *comp* =yes |
| Step 8 |  | *d* =d3  *r* =max  *ttt* =norm  *ctrl* =max  *comp* =yes |
| Step 9 |  | *Stop* |
