# Supplementary material for: The development of an adaptive upper-limb stroke rehabilitation robotic system
Source: J Neuroeng Rehabil. 2011 Jun 16;8:33. doi: 10.1186/1743-0003-8-33 (PMC3152889; doi:10.1186/1743-0003-8-33)
Supplement: Additional file 4 — POMDP simulation example 2. This file shows the simulation steps of example 2. [file 1743-0003-8-33-S4.DOC]

# Simulation steps of Example 2

| Decision  Steps | Belief state | Actions &  observations |
| --- | --- | --- |
| Step 1 |  | *d*=d1  *r*=none  *ttt*=norm  *ctrl*=max  *comp*=no |
| Step 2 |  | *d*=d2  *r*=none  *ttt* =norm  *ctrl* =max  *comp* =no |
| Step 3 |  | *d*=d3  *r*=none  *ttt* =none  *ctrl* =min  *comp* =no |
| Step 4 |  | *d*=d3  *r*=none  *ttt* =none  *ctrl* =min  *comp* =no |
| Step 5 |  | *d*=d3  *r*=none  *ttt* =none  *ctrl* =none  *comp* =yes |
| Step 6 |  | *Stop* |
